# Bioactive and biodegradable cotton fabrics produced via synergic effect of plant extracts and essential oils in chitosan coating system

**DOI:** 10.1038/s41598-024-59105-4

**Published:** 2024-04-12

**Authors:** Bolesław Szadkowski, Magdalena Śliwka-Kaszyńska, Anna Marzec

**Affiliations:** 1 Institute of Polymer and Dye Technology, Faculty of Chemistry, Lodz University of Technology, Stefanowskiego 16, 90-537 Lodz, Poland; 2 Department of Organic Chemistry, Chemical Faculty, Gdansk University of Technology, Narutowicza 11/12, 80-233 Gdansk, Poland

**Keywords:** Materials science, Composites, Materials chemistry

## Abstract

Functional antibacterial textile materials are in great demand in the medical sector. In this paper, we propose a facile, eco-friendly approach to the design of antibacterial biodegradable cotton fabrics. Cotton fiber fabrics were enhanced with a chitosan coating loaded with plant extracts and essential oils. We employed Fourier-transform infrared (FTIR) and X-ray photoelectron spectroscopy (XPS), UV–Vis spectrophotometry, optical microscopy, scanning electron microscopy (SEM), and thermogravimetric analysis (TGA) to characterize the color, structure, and thermal properties of the modified fabrics. The fabrics were found to effectively induce growth inhibition of Gram-positive and Gram-negative bacteria, especially when a synergic system of aloe vera extract and cinnamon essential oil was applied in the coating formulation. Additionally, we observed significant color and weight changes after 5, 10, and 20 days in soil biodegradability tests. Given the straightforward modification process and the use of non-toxic natural materials, these innovative bio-based and biodegradable cotton fabrics show great promise as protective antimicrobial textiles for healthcare applications.

## Introduction

Bacterial contamination of textile fabric surfaces is a common hazard in the medical health care sector (hospitals, medical centers, etc.). Microbial shedding occurs when patients come into contact with textiles, spreading infections and cross-infections. Therefore, there is significant interest in the use of bioactive textiles to prevent the migration of pathogenic bacteria between patients and healthcare professionals^1^. The COVID-19 pandemic underscored the need for new effective antimicrobial materials that are also eco-friendly within the medical sector. Cotton fabrics are the most common textile materials used for medical and biomedical purposes (e.g. surgical clothing, surgical covers, bedding). Cotton exhibits very good mechanical properties, large surface area, high porosity, and biodegradability. Because of its good chemical reactivity, it is also easy to modify^2^. However, cotton has poor resistance against microbial growth (i.e., fungi and bacteria), which significantly limits its use in more advanced medical applications^3^.

Several approaches have been proposed for improving the antibacterial properties of cotton-based materials. These include chemical coatings, stabilization of metal nanoparticles, and surface plasma treatment^4–6^. Functionalization with chemical coatings has emerged as a facile and effective strategy for the design of multifunctional textile fabrics with antimicrobial properties. The extensive literature on this topic highlights the benefits of functionalization of cotton-based coatings with chemical coatings, such as low-cost, high homogeneity, good affinity, and the simple process of modification^7,8^. However, the traditional chemical additives used in commercial textiles are often non-biodegradable. This can result in serious environmental pollution and consequently affect human health.

Chitosan biopolymer coatings offer a promising alternative. This is due to the unique properties of chitosan, which include not only biodegradability and biocompatibility, but also film-forming capacity, non-toxicity, and antimicrobial activity^9–11^. The combination of chitosan coating with functional eco-friendly additives could add further benefits, and is attracting much attention from researchers. Many plant extracts and essential oils antibacterial, antifungal, biodegradable, and safe for human health^12^. These natural substances are often used in various applications, including in traditional and alternative medicine, personal care products, and as natural preservatives. Essential oils are rich in phenolic compounds (such as carvacrol and thymol), which provide high antibacterial, phytotoxic, and insecticidal activity^13^. For instance, Ibrahim et al.^14^ demonstrated that different essential oils (such as lavender, clove, aloe vera or cinnamon oils) enhanced the antimicrobial properties of cotton fabrics modified with monochlorotriazinyl β-cyclodextin (MCT-βCD). Plant extracts, similarly, to essential oils, are also known for their rich composition of bioactive compounds, although their specific constituents can vary widely depending on the plant source. These extracts often contain a diverse array of phytochemicals such as flavonoids, tannins, alkaloids, terpenoids, and phenolic acids. The presence of these active ingredients caused that natural dyes and pigments can also have a positive effect on the biocidal properties of textile materials^15,16^.

Ibrahim et al.^17^ developed the procedure of simultaneous hand-building, anti-bacterial protecting and UV-blocking of cotton fabric using different finishing/coating formulations based on biopolymers, eco-friendly ester crosslinking system and metal nanoparticles. Javid et al.^18^ studied how the concentrations of chitosan, essential oil, and surfactant affected the antibacterial properties of modified cotton fabrics. The antibacterial activity of the fabrics increased with increasing concentrations of both eucalyptus and sandalwood oils in the formulation. Kumar et al.^19^ demonstrated that loading chitosan with frankincense oil can have positive impact on the antioxidant activity, antibacterial fragrance, and mosquito repellent activity of cotton fabric materials, while also enhancing flame retardancy. Chitosan/herbal composites can also be used to create antimicrobial cotton fabrics. For instance, Chandrasekar et al.^20^ used chitosan compositions with two medicinal plant extracts from Senna auriculata and Achyranthes aspera in a ratio of 1:1. The formulation increased the durability and antibacterial activity of the finished cotton fabric, which could potentially be used for medical textile materials. Good antibacterial activity was also observed for other cotton fabrics modified with different plant extracts, such as Aloe vera^21,22^, Azadirachta indica^23^, Ocimum sanctum^24^, and Piper betle^25^. However, there have been no significant studies on antimicrobial cotton fabrics modified with chitosan coatings containing both plant extracts and essential oils. Multi-component coatings can influence a range of properties simultaneously, including color, fragrance, and antibacterial activity. Moreover, the bioactive ingredients may have synergistic effects.

Here, we present bioactive and biodegradable cotton fabrics designed for applications as single-use medical textiles. We study the effects of functionalization with chitosan-based coatings on the structure, color, thermal stability, biodegradability and anti-microbial activity of cotton fabric. Due to the synergic effects between the chitosan biopolymer, natural plant extracts, and essential oils present in the coating system, the modified cotton fabrics showed high biological activity. Importantly, these coatings are also biodegradable, which is desirable from an environmental standpoint. The design of single-use antibacterial textiles would be a significant step towards solving the critical issue of managing the large amounts of contaminated waste produced by the medical industry. This straightforward and eco-friendly approach paves the way for developing antibacterial coating systems for cotton fabrics, with potential applications in various medical sectors where textile materials are essential.

## Experimental details

### Materials

**Figure 1:**
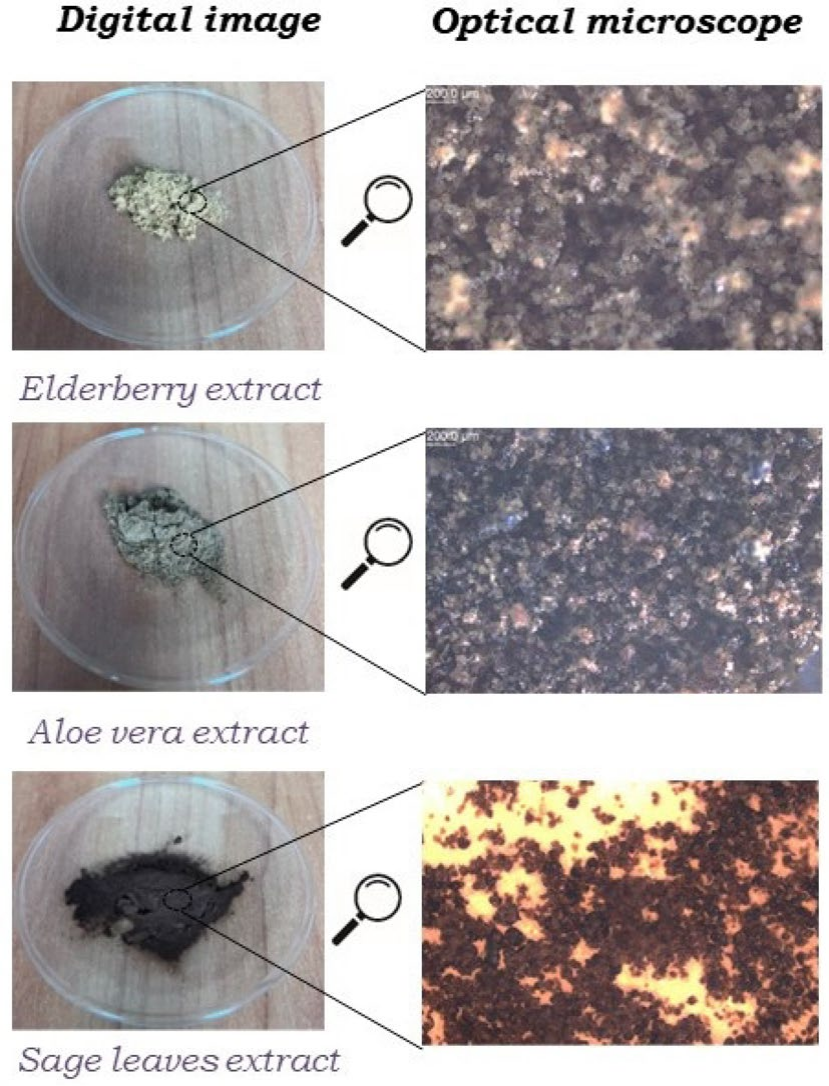
Images of the plant extracts used in chitosan-based formulations.

Chitosan with high-molecular weight (310,000–375,000 Da, degree of deacetylation > 75%) and the viscosity of 800–2000 cP as well as citric acid (99%) were purchased from Sigma–Aldrich Chemical Co (Steinheim, Germany). Commercial sourced cotton fabric with grammage of 124 m^SPS:refid::bib22^/g was supplied by Matimpex-Importer PPHU (Lodz, Poland). Lavender and cinnamon essential oils were provided by Torimex-Chemicals (Konstantynow Lodzki, Poland). Elderberry (*Sambucus nigra L.*), sage leaves (*Salvia officinalis L.*) and aloe vera (*Aloe barbadensis Miller*) powder extracts were kindly provided by Safic-Alcan (Fig. 1). All chemical reagents were used in the study without further purification. The bacteria *S. aureus* ATCC 6538, *E. coli* ATCC 10536, *P. fluorescens* ATCC 13525, and *B. subtilis* ATCC 6633, as well as the fungi *A. niger* ATCC 16404, and *C. albicans* ATCC 10231 were used to carry out the antimicrobial testing and were bought from the American Type Culture Collection by Argenta (Poznan, Poland).

### Modification procedure

Firstly, the chitosan coating formulation has been prepared by dissolving chitosan in a solution of citric acid in water at the temperature of 50 ℃. Subsequently, the appropriate amount of plant extract powder (1 g) was added, and the resulting reaction system was subjected to intense mechanical stirring until a clear, homogeneous solution was obtained. Then, the resulting homogeneous solution was cooled to room temperature, and essential oil (1 g) was added dropwise thereto. The reaction system was then mixed under mechanical stirring for the next 1 h. Cotton fabrics have been subjected to alkaline scouring and bleaching pre-treatment following the procedure described elsewhere^26^. Subsequently, the as-prepared cotton fabrics were entered in the prepared solutions and kept for 30 min at room temperature in a material-to-liquor ratio of 1:20. The samples were finally washed several times with deionized water and dried in the room temperature for the next 24 h. The following samples were obtained as a result of the above-described modification: cotton fabric modified with chitosan/elderberry extract/(CF/CS/EB), cotton fabric modified with chitosan/sage leaves extract/(CF/CS/SL), cotton fabric modified with chitosan/aloe vera extract/(CF/CS/AV), cotton fabric modified with chitosan/aloe vera extract/lavender essential oil (CF/CS/AV/LEO), cotton fabric modified with chitosan/aloe vera extract/cinnamon essential oil (CF/CS/AV/CEO), cotton fabric modified with chitosan/sage leaves extract/lavender essential oil (CF/CS/SL/LEO), cotton fabric modified with chitosan/elderberry extract/lavender essential oil (CF/CS/EB/LEO).

### Methods

The color of the cotton fabrics modified with chitosan-based coatings was determined based on the CIE 15:2004 standard with the use of a CM-3600d spectrophotometer (Konica Minolta Sensing, Japan). The color characteristic of the materials was described following the CIE-Lab system (a—red-green coordinate, b—yellow-blue coordinate, L—lightness coordinate). The total color change (ΔE) parameter was calculated based on the Eq. (1) ^27^:

1 $$\Delta E=\sqrt{{(\Delta L)}^{2}+{(\Delta a)}^{2}+{(\Delta b)}^{2}.}$$

Scanning electron microscopy (SEM) analysis of the fabrics was performed on a Leo 1530 Gemini scanning electron microscope (SEM, Zeiss, ULTRA Plus, Oberchoken, Germany) operating at an accelerating voltage of 15 keV. The morphological characteristics of the modified cotton fabrics was also performed using an Optatech optical microscope (Optatech, Warsaw, Poland) coupled with a Leica MZ 6 camera (Wetzlar, Germany) and OptaView software (Optatech, Warsaw, Poland). The thermal stability of studied materials was evaluated by thermogravimetric analysis (TGA). For TGA measurements, an approximately 10 mg of the samples were placed in alumina crucibles and heated from 25 to 800 °C under argon (flow 50 mL/min) at a rate of 10 °C/min (Mettler Toledo Thermobalance, TA Instruments, New Castle, DE, USA). Fourier transform infrared spectroscopy (FTIR) was carried out using a ThermoScientific Nicolet 6700 FTIR spectrometer equipped with diamond Smart Orbit ATR sampling accessory (Waltham, MA, USA). FTIR measurement was performed within the spectral range of 4000–400 cm^−1^ with 64 scans. The chemical composition of the modified cotton fabrics was inspected using X-ray photoelectron spectroscopy (XPS) analysis. XPS measurements were performed using an X-ray photoelectron hemispherical spectrometer (Argus Omicron Nano-Technology) with a Mg–Kα source of X-ray and anode operated at 15 keV, 300 W. The analysis was conducted under an ultra-high vacuum at room temperature, with pressure below 1.1 × 10^−8^ mbar. The biological properties (antibacterial) measurements of the modified cotton fabrics were conducted following the PN-EN ISO 20645:2006 (bacteria) and PN-EN ISO 14119:2005 (fungi) standards. Two parallel rows of inoculum with density of 1–2 × 108 CFU/ml (bacteria) or 12 × 106 CFU/ml (fungi) were applied to plates with MEA (fungi) or TSA (bacteria) medium. The samples with dimensions of 60 × 25 mm were placed perpendicular to the growth line and incubated at 30 ℃, except for the samples with *E. coli*, *S. aureus*, and *C. albicans* which were incubated at 37 ℃ for 48 h for bacteria and yeasts and 96 h for mold. After incubation, the growth was observed, and zones of growth inhibition were measured according to a scale:

- Very good activity (+++)—growth inhibition around and under the sample/inhibition zone > 1 mm.
- Good activity (++)—growth inhibition around and under the sample/inhibition zone < 1 mm or growth inhibition under the sample/lack of inhibition zone.
- Low activity (+)—low growth under the sample/lack of inhibition zone.
- Lack of activity (−)—growth under the sample/lack of inhibition zone.

Soil biodegradation experiments for studied cotton fabrics were performed following the PN-EN ISO 846 standard. The studied specimens were placed in universal soil in a climatic chamber (MEMMERT type HPP 108, Memmert GmbH + Co. KG, Schwabach, Germany) for 5, 10, and 20 days at 30 °C, with relative humidity of air WWP = 80%. Subsequently, the samples were removed from the soil, rinsed gently with tap water with soap and the progress of soil biodegradation was determined based on the weight loss of samples (Eq. 2):

2 $${W}_{t}\left(\%\right)= \frac{({W}_{0}-{W}_{t})}{{W}_{0}}\times 100,$$

where W_t_ (%) represents the percentage of weight loss after t days of biodegradation, W_0_ (g) is the initial weight and W_t_ (g) is the weight of the dry fabric after t days of biodegradation.

## Results and discussion

### Color and structure of cotton fabrics

**Figure 2:**
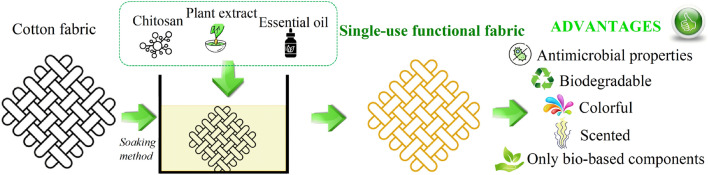
Diagram presenting graphically the concepts of the research on new single-use functional fabric.

The main aim of this work was to design new single-use textile materials by enhancing cotton fabrics with a chitosan coating containing various plant-based additives. We investigated the effects of different chitosan-coatings on the morphology and color, as well as thermal, antimicrobial, and biodegradable properties of the designed textile materials (Fig. 2).

The successful functionalization of the CF using chitosan-based coatings has been confirmed using FTIR and XPS spectroscopy methods (Figs. S1 and S2, supplementary materials). The FTIR study revealed that modification of CF with chitosan contributed to the appearance of new double absorption peak with the maximums at 1550 cm^−1^ and 1650 cm^−1^, respectively. Such results have already been reported for cotton fabrics covered with different functional chitosan coatings^21^. Moreover, XPS analysis showed that the modification of cotton fabric using a chitosan coatings contributed to an increase in the nitrogen content from 0.5% (CF control sample) up to 1.2% and 1.1% for CF/CS and CF/CS/AV/CEO samples, respectively (Table S1, supplementary materials). Obviously, the amount of nitrogen in sample CF/CS belong to the amino groups introduced onto the cotton fibers by the adsorption of chitosan biopolymer. These results prove that the chitosan coating systems have been effectively bonded to the cotton fabric. Because of the presence of hydroxyl groups and the arrangement of the molecular chains, it is thought that the interaction mechanism between chitosan-based coatings and cellulose from cotton involves the formation of hydrogen bonds between the –OH groups in cellulose and the amino or hydroxyl groups in chitosan biopolymer. The chemical and molecular structures of cellulose and chitosan are similar, facilitating a strong affinity between the two biopolymers. Therefore, the primary intermolecular interactions between cellulose and chitosan are most probably based on hydrogen bonds and Van der Waals forces^28^.

Modifying cotton fabrics with the chitosan-based coatings resulted in color changes compared to the raw textile material. The color depended mainly on the type of plant ex-tract used in the chitosan coating. However, all obtained materials exhibited a light brown-like color. Digital, optical microscopic, and scanning electron microscopic (SEM) images of the cotton fabrics are shown in Table 1. Colorimetric results from spectrophotometric measurements are presented in Table 2.

**Table 1:**
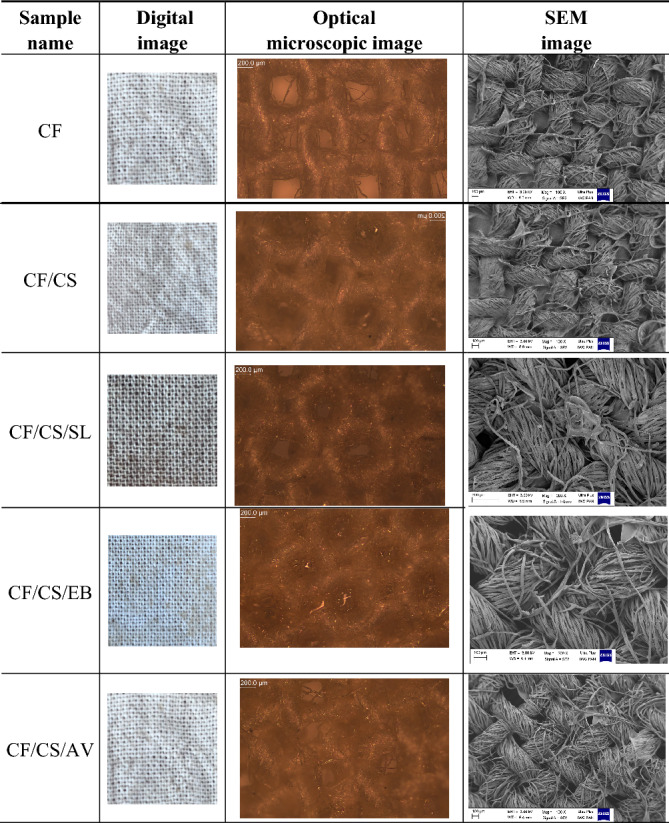
Images of the modified cotton fabrics.

**Table 2:** Color coordinates of studied CF samples determined in spectrophotometric measurements following the CIE Lab color space system.

Sample name	ΔE	L*	a*	b*
CF	–	91.88	0.27	14.59
CF/CS	9.17	85.20	1.52	20.71
CF/CS/SL	34.59	60.57	8.63	26.51
CF/CS/EB	16.67	79.97	5.35	25.08
CF/CS/AV	16.52	81.13	3.62	26.68
CF/CS/SL/LEO	32.77	64.09	9.15	29.50
CF/CS/EB/LEO	24.00	75.21	7.32	30.34
CF/CS/AV/LEO	17.67	80.69	3.87	27.78
CF/CS/AV/CEO	17.11	80.12	3.77	26.49

As can be seen from the images in Table 1, all the cotton fabrics showed similar morphologies. The raw CF sample exhibited a typical longitudinal fibril structure, with smooth surfaces. The gaps between strands were in the range of 50–200 microns in size, which correlates well with the previous reports on raw cotton fabrics^29,30^. Modifying the cotton fabrics with different chitosan-based coatings did not contribute to significant changes in terms of morphology. This means that the designed bio-formulations formed a uniform, transparent, and homogeneous layer on the surface of cotton fabric.

The application of coatings containing colorful plant extracts produced modified CF samples with different color characteristics. Table 2 shows the L^*^a^*^b^*^ and ∆E values for the coated cotton fabrics. As expected, the modification of CF with chitosan coatings resulted in significant color changes. This was clearly reflected in the L^*^a^*^b^*^ and ΔE parameters for all the studied materials. For instance, the CF sample coated with raw chitosan (CF/CS) showed ∆E = 9.17 (clearly visible change in color to an unexperienced observer), while materials modified with coatings containing colorful plant extracts exhibited more pronounced ∆E parameters, such as 16.52, 16.67, and 34.59 for CF/CS/AV, CF/CS/EB, and CF/CS/SL, respectively. In addition, the brightness parameter L^*^ decreased in the case of CF samples after modification with various chitosan coatings. The most significant reduction in brightness was observed for the CF/SL sample reaching an L^*^ value of 64.09 (in comparison, the L^*^ for neat CF was 91.88). This was obviously due to the dark brown color of the SL extract present in the chitosan formulation. Depending on the plant extract used for modification, the a^*^ and b^*^ parameters of the CF-coated systems also changed. So, appropriate selection of the type and concentration of colorful plant extracts may be useful for tuning the color of cotton fabrics modified with chitosan coatings.

### Antibacterial properties of cotton fabrics

**Figure 3:**
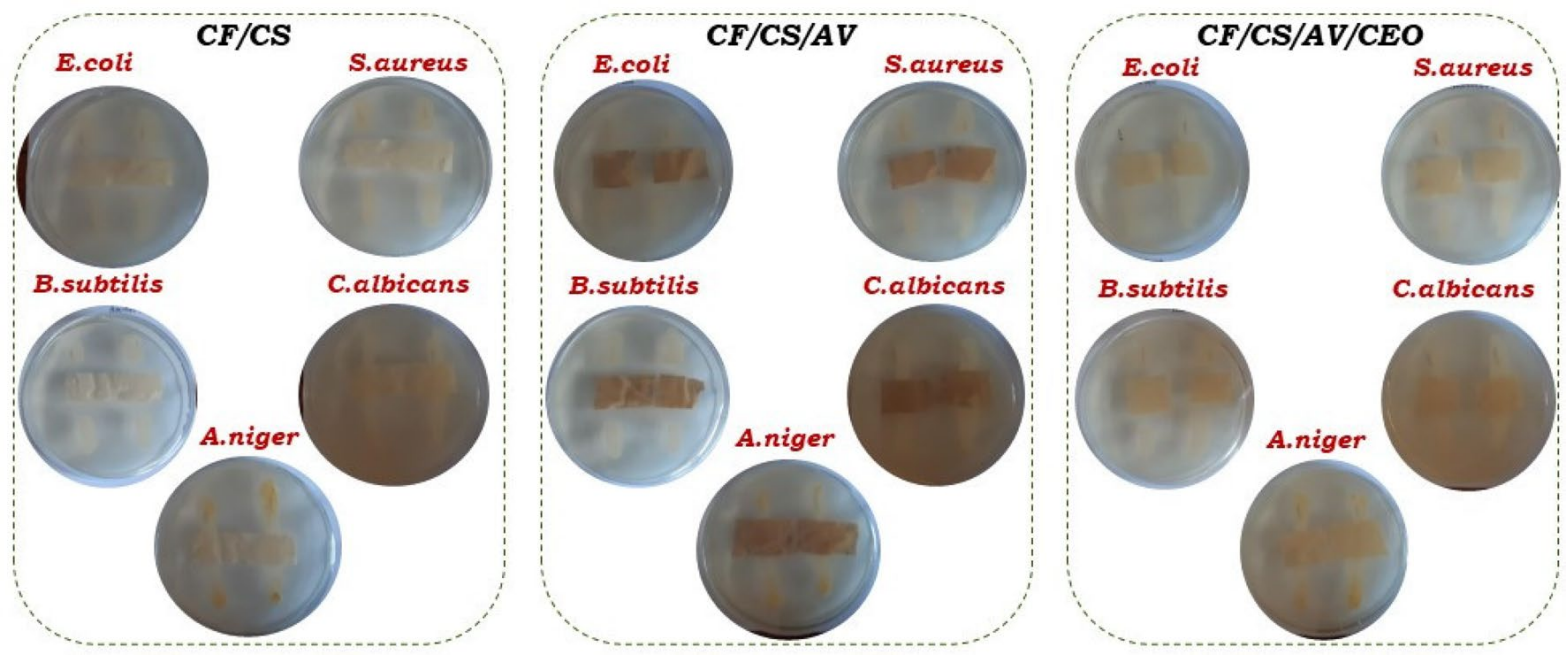
Illustration of the antimicrobial effect of the investigated cotton fabrics modified with different chitosan-based coatings.

Textile materials for medical applications must meet stringent requirements to protect patients against pathogenic microorganisms. Chitosan-based coatings offer a useful means for endowing textiles with antibacterial properties. This is attributed to chitosan’s wide spectrum of activity and high killing rate against gram-positive and gram-negative bacteria^31^. We investigated the antimicrobial effects of newly developed chitosan coatings containing different bioactive extracts and essential oils. The modified cotton fabrics with chitosan-based coatings were tested against *E. coli*, *S. aureus*, *B. subtilis*, *C. albicans*, and *A. niger*. The results are presented in Table 3 and Fig. 3.

**Table 3:** Antimicrobial activity of the designed cotton fabric materials.

Sample name	*Escherichia coli*	*Staphylococcus aureus*	*Bacillus subtilis*	*Candida albicans*	*Aspergillus niger*
CF (control sample)	–	–	–	–	–
CF/CS	+++	+++	+++	–	+
CF/CS/SL	+++	+++	+++	–	+
CF/CS/EB	+++	+++	+++	+	+
CF/CS/AV	+++	+++	+++	+	+
CF/CS/SL/LEO	+++	+++	+++	+	++
CF/CS/EB/LEO	+++	+++	+++	+	++
CF/CS/AV/LEO	+++	+++	+++	+	++
CF/CS/AV/CEO	+++	+++	+++	++	++

+++ Very good activity; ++ good activity; + low activity; – lack of activity.

As can be seen in Table 3, untreated cotton fabric (CF) showed no antimicrobial activity (–) against any of the studied microorganisms. As expected, modification with chitosan coating endowed the textile material with very high antibacterial activity (+++) to *E. coli*, *S. aureus*, and *B. subtilis*. The CF/CS sample also showed some inhibitive activity against *A. niger*, suggesting additional anti-fungicidal properties. The antimicrobial properties of the CF/CS sample were evidenced by the visible inhibition zone between the fabric and the tested microorganisms, as illustrated in Fig. 3. The positive effects of chitosan coating on the antimicrobial activity of cotton fabrics have been reported previously by other authors^32–34^. For instance, Staneva et al.^35^ reported recently that chitosan coating modified with benzaldehyde and crosslinked with glutaraldehyde can provide an effective antimicrobial coating for cotton fabrics. The authors found that chemical modification of chitosan with benzaldehyde increased its hydrophobicity, resulting in improved antimicrobial activity. In another study, Wang et al. produced durable antimicrobial cotton fabrics modified with carboxymethyl chitosan and quaternary ammonium salts^36^. The bacteriostatic reduction rate (BR) of the modified cotton fabric against *S. aureus* and *E. coli* was above 99.9%, and the BR value remained above 99.9% even after 120 laundering cycles.

Two primary mechanisms underlie the antibacterial effect of the chitosan biopolymer. Firstly, the amine groups in chitosan can become positively charged (NH_3_^+^), allowing them to interact with and disrupt the cell walls of microorganisms, thereby inhibiting their growth^37^. The second mechanism involves the penetration of chitosan into the cells, where it absorbs anionic substances crucial to cell structure, consequently interfering with the cell’s normal functions^37,38^. When considering high-molecular weight chitosan, it is believed that it can form a dense biopolymer film on the surface of the bacterial cell, blocking the exchange of nutrients by covering porins resulting in the microbial cell death^39^.

The incorporation of sage leaf (*Salvia officinalis L*.) extract into the chitosan coating did not changed its antibacterial properties. Elderberry (*Sambucus nigra L*.) and aloe vera (*Aloe barbadensis Miller*) extracts provided additional weak protection against *C. albicans*. Importantly, enriching the chitosan/plant extract coating formulations with essential oils resulted in systems with more pronounced bioactivity. The synergistic effects of plant extracts and lavender essential oil resulted in CF/CS/SL/LEO, CF/CS/EB/LEO, CF/CS/AV/LEO, and CF/CS/AV/LEO samples with very good bioactivity (+++) against *E. coli*, *S. aureus*, and *B. subtilis*, low bioactivity (+) against *C. albicans*, and good bioactivity (++) against *A. niger*. Replacing the lavender essential oil with cinnamon essential oil resulted in improved biological activity against *C. albicans*, from low activity (+) for CF/CS/AV/LEO to good activity (++) for CF/CS/AV/CEO.

### Thermal- and bio-degradation characteristics

**Figure 4:**
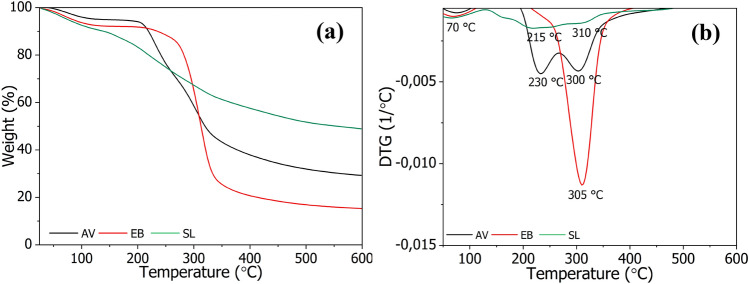
TGA (**a**) and DTG (**b**) curves of the studied plant extracts.

Thermogravimetric (TGA/DTG) profiles for AV, EB, and SL plant extracts in nitrogen atmosphere are presented in Fig. 4. All TGA curves show gradual decomposition, starting with loss of the water content at around 70 ℃^35^. Generally, the main thermal decomposition stage of the pure plant extracts occurs between 200 and 350 ℃. The AV and SL extracts show two thermal decomposition events at around 200 ℃ and 300 ℃, which can be attributed to the decomposition of hemicellulose, cellulose, and lignin. Similar TGA/DTG results have been reported previously for aloe vera extracts^40,41^. Meanwhile, the EB extract exhibited its main decomposition DTG peak at 305 ℃, suggesting higher thermal stability compared to AV and SL. Nonetheless, based on the TGA/DTG results it can be concluded that the thermal stability of the tested extracts is satisfactory from the point of view of their application in chitosan coatings for textile materials.

**Figure 5:**
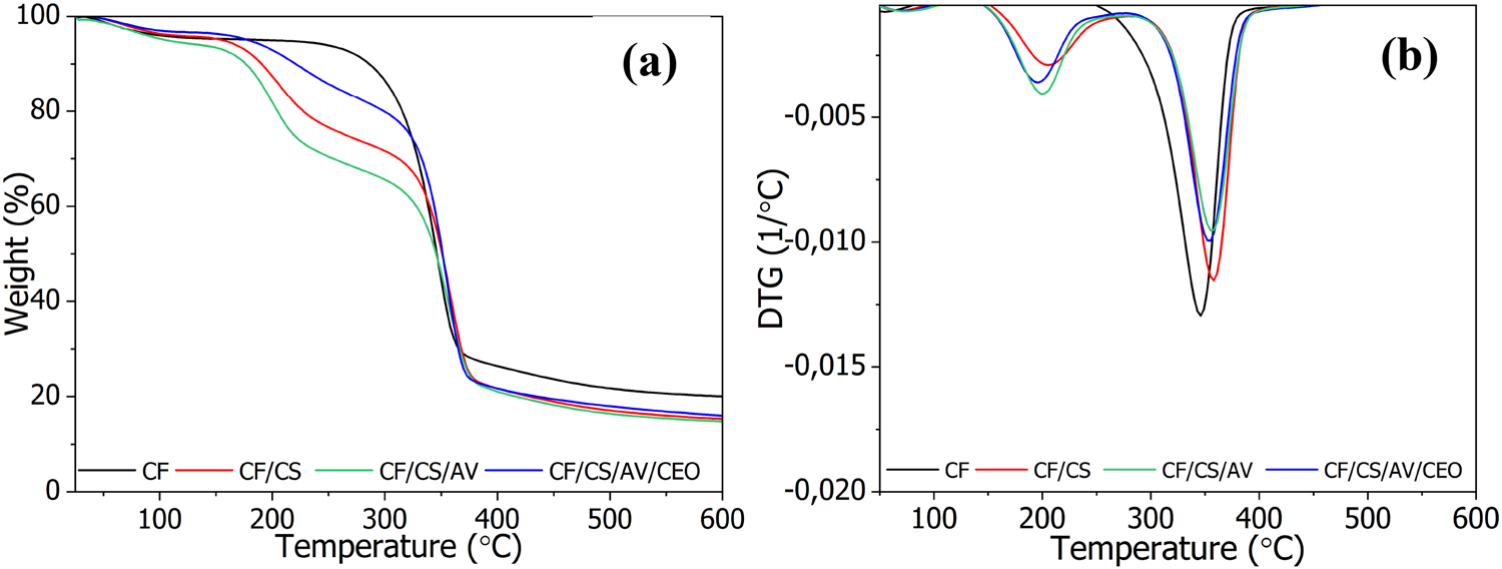
TGA (**a**) and DTG (**b**) curves of the studied cotton fabrics.

The thermal decomposition behavior of the designed CF systems was investigated using Thermogravimetric analysis (TGA) under nitrogen air conditions. Thermograms and corresponding thermal decomposition temperatures are presented in Fig. 5 and Table 4.

**Table 4:** Thermal decomposition temperatures and char residues of the selected studied cotton fabric samples determined in TGA measurements.

Sample name	T_05%_	T_10%_	T_20%_	Char residue at 600 °C [%]
CF	196	283	316	20
CF/CS	157	208	229	15
CF/CS/AV	106	175	205	14
CF/CS/AV/CEO	175	220	301	16

T_05,10,20%_—thermal decomposition temperature for 5, 10, and 20% reduction in sample weight.

As can be seen in Fig. 5 and Table 4, the control CF sample began to decompose at 196 °C (T_05%_) and exhibited a one-step degradation process from about 200–400 °C. This process is attributed to two competitive processes involving glycosyl units. After modification with chitosan coatings, the cotton fabrics showed three-step weight loss and lower T_05%_, T_10%_, and T_20%_ values compared to the control sample. Similar results have been reported previously for cotton fabrics coated with chitosan, sodium phytate, and hydrolyzed APTES^42^. It was concluded that the reduced thermal stability of the modified CF materials was due to the release of adsorbed water from the modifying agents. Importantly, the raw CF sample formed thermally stable aromatic char residues of about 20% at 600 °C. Coating the CF with chitosan-based formulations reduced the char residue to around 14–16%.

**Figure 6:**
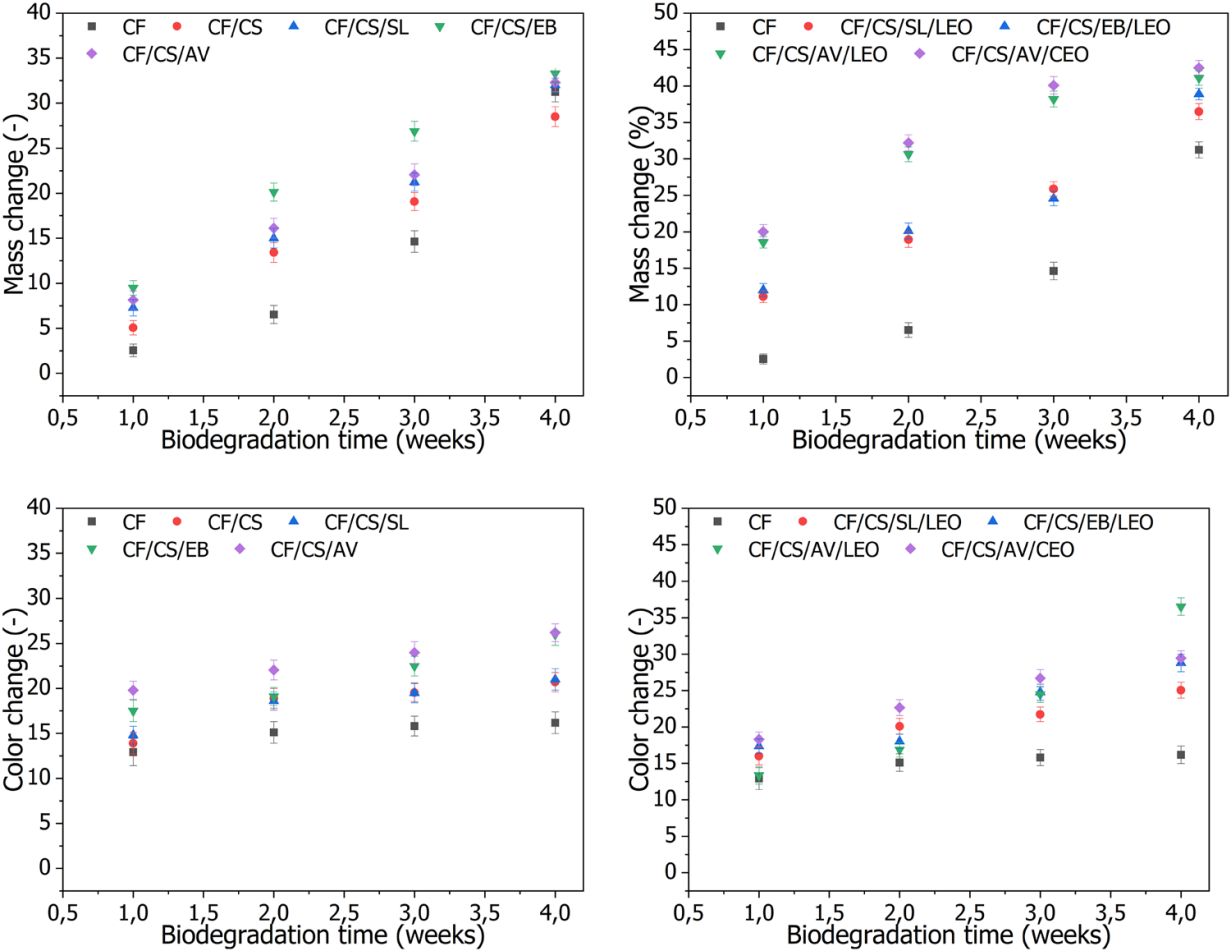
Mass and color change of the fabric samples subjected to the soil biodegradation test.

According to the literature, cotton fabrics are promising candidates for preparing biodegradable, single-use textile products for the medical sector^43,44^. Therefore, the last stage of our research was to analyze the impact of the chitosan coating containing natural additives on the biodegradability of the cotton fabrics. The modified CF materials were subjected to soil biodegradation experiments. Changes in their physical appearance and weight were monitored after 1, 2, 3, and 4 weeks. The color change and weight loss of the fabrics after degradation in soil are presented in Fig. 6. Digital images of the samples are shown in Table 5.

**Table 5:**
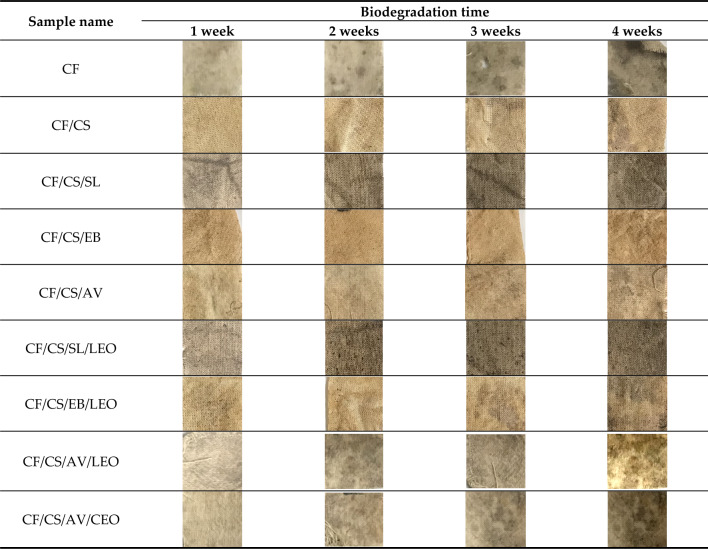
Digital images of the fabric samples subjected to the soil biodegradation test.

Figure 6a,b present the cumulative percentage weight loss of the studied CF samples buried in soil, as a measure of the degree of degradation. Based on the results, microorganisms in the soil quickly infiltrated the studied CF samples, as evidenced by significant weight loss and color changes (Fig. 6). The weight loss of the studied materials increased progressively over time. The CF samples were seriously biodegraded within 4 weeks, with 30–45% weight loss. Clear changes in the physical appearance of the biodegraded CF materials are also visible in the digital images in Table 5. In general, modifying the raw cotton fabric with chitosan-based coating led to more pronounced changes in the weight and color of the fabrics. This is in good agreement with the results of our previous research on functional cotton fabrics modified with chitosan coatings^45^. The improved biodegradability of the CF coated with chitosan formulation may be explained by the hydrophilic nature of the biopolymer, which makes it more susceptible to biodegradation^43^. The presence of bio-additives (extracts and essential oils) in the chitosan coating did not result in significant differences in biodegradation ability. However, the highest percentage weight losses, at 42.5% and 41.2% after 4 weeks, were obtained for the CF/CS/AV/CEO and CF/CS/AV/LEO samples, respectively. These samples also exhibited the most visible color changes during the soil biodegradation test. After 4 weeks, the raw CF sample reached ΔE = 16.18, while CF/CS/AV/CEO and CF/CS/AV/LEO samples showed ΔE = 29.47 and ΔE = 36.52, respectively. All tested samples were characterized by pronounced color changes (ΔE), with values for this parameter above 10 after just 1 week (where ΔE above 3 indicates clear and easy to recognize color changes for the observer). These changes in color performance constitute the first solid evidence biodegradation. Overall, the results for weight loss and color change confirmed that the designed eco-fabrics showed not only high biocidal effectiveness but also good biodegradability, making them a promising candidate for the production of sustainable textile materials.

## Conclusions

In this study, we have demonstrated a novel design for bioactive and biodegradable cotton fabrics functionalized with chitosan-based coatings containing plant extracts and essential oils. Importantly, the modification process does not significantly affect the inherent attractive attributes of cotton fabric.

Cotton fabrics were modified with chitosan-based coatings containing various plant-based additives. The modified fabrics were analyzed in terms of their morphology, color, thermal stability, biodegradability, and antibacterial activity. The application of synergic systems of plant extracts and essential oils enhanced the antibacterial functionality of the chitosan coating formulations and gave a pleasant smell to the textile material. The addition of plant extracts and essential oils in the chitosan coating formulation clearly improved its antibacterial effectiveness against *E. coli, B. subtilis, S. aureus, C. albicans*, and *A. niger*. The most promising results were obtained for the coating containing chitosan, aloe vera extract, and cinnamon essential oil. In biodegradation tests, both the control cotton fabric and modified samples disintegrated into pieces after 4 weeks of burial in soil. Significant loss of mass and color changes indicated that all samples were biodegraded. In conclusion, the synergistic use of chitosan biopolymer along with plant-based extracts and essential oils represents a promising strategy for developing bioactive and biodegradable textile materials. These innovative textiles hold great potential for various applications in the medical healthcare sector.

### Supplementary Information

Supplementary Information.

## Data Availability

The data generated during this study is available from the corresponding author on reasonable request.
