# Supplementary material for: Bioactive and biodegradable cotton fabrics produced via synergic effect of plant extracts and essential oils in chitosan coating system
Source: Sci Rep. 2024 Apr 12;14:8530. doi: 10.1038/s41598-024-59105-4 (PMC11014983; doi:10.1038/s41598-024-59105-4)
Supplement: Supplementary file 1 — Supplementary Information. [file 41598_2024_59105_MOESM1_ESM.docx]

**Supplementarny materials.**

Bioactive and biodegradable cotton fabrics produced via synergic effect of plant extracts and essential oils in chitosan coating system

# Bolesław Szadkowski^1,*^, Magdalena Śliwka-Kaszyńska^2^, Anna Marzec^1,*^

^1^Institute of Polymer and Dye Technology, Faculty of Chemistry, Lodz University of Technology, Stefanowskiego 16, 90-537 Lodz, Poland

^2^Department of Organic Chemistry, Faculty of Chemistry, Gdansk University of Technology, Narutowicza 11/12, 80-233 Gdansk, Poland

^*^[boleslaw.szadkowski@p.lodz.pl](mailto:boleslaw.szadkowski@p.lodz.pl) and [anna.marzec@p.lodz.pl](mailto:anna.marzec@p.lodz.pl)


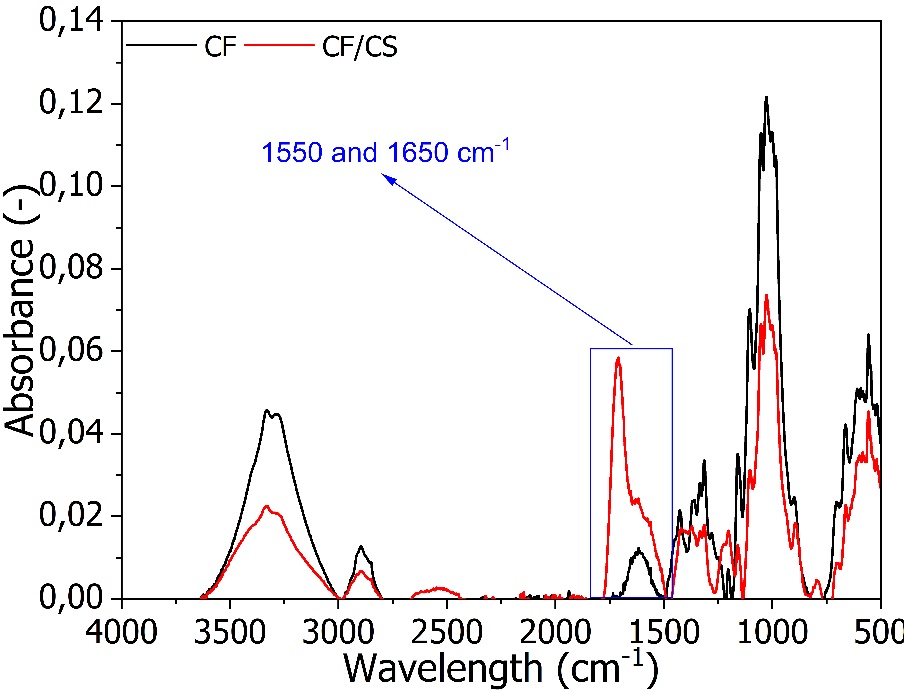


**Figure S1.** FTIR spectra of the studied cotton fabrics.


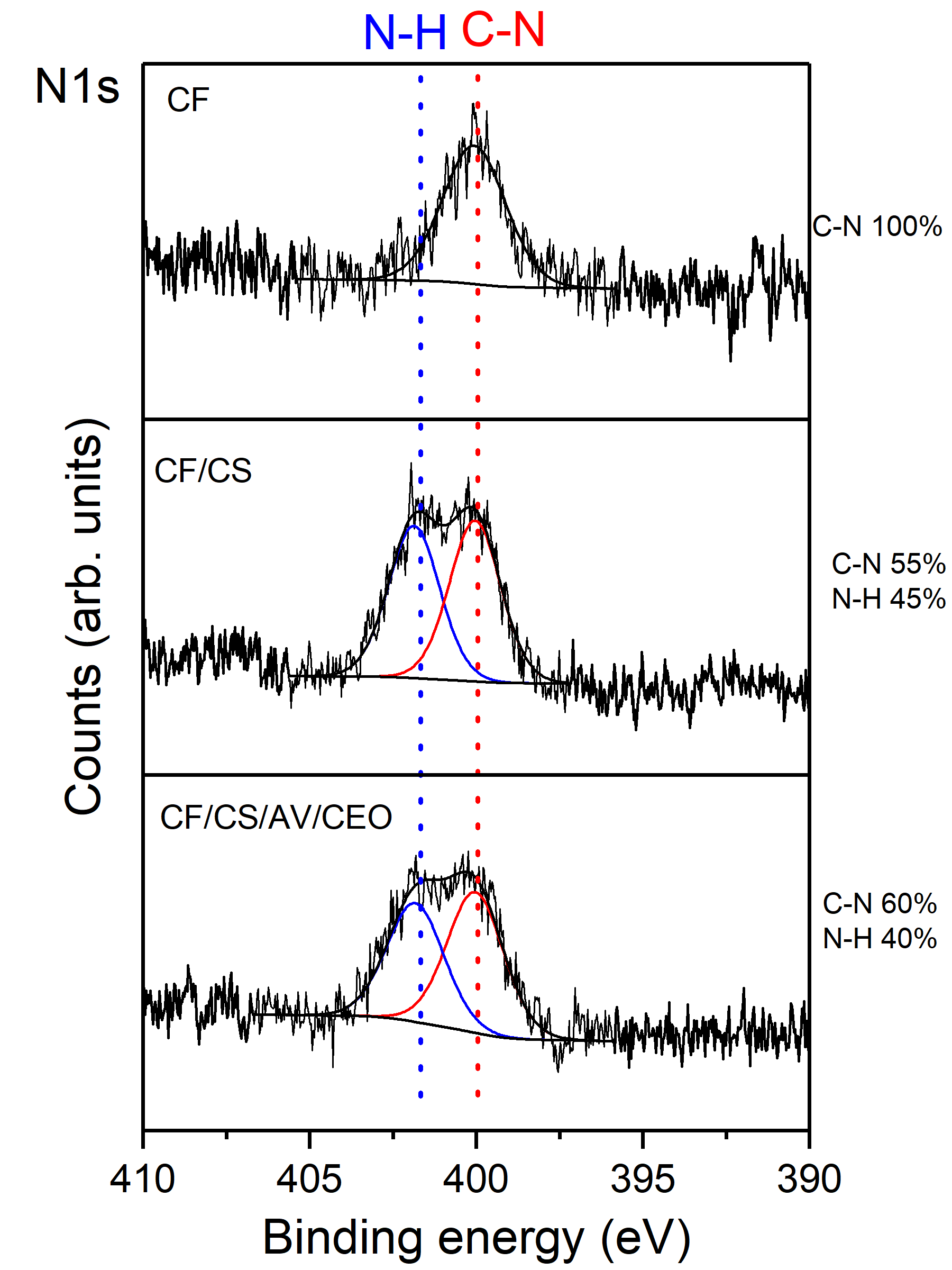


**Figure S2.** N1s XPS spectra of the studied cotton fabrics.

| **Sample name** | ***%N*** |
| --- | --- |
| CF | 0.5 |
| CF/CS | 1.2 |
| CF/CS/AV/CEO | 1.1 |

**Table S1.** Nitrogen elemental analysis of the studied cotton fabrics obtained from XPS experiment.
